# Rupture of the Extensor Digitorum Tendon of the Fifth Toe Caused by the Use of Press-Tack Needle Acupuncture During Sports Activity: A Case Report

**DOI:** 10.7759/cureus.112431

**Published:** 2026-07-10

**Authors:** Sakurako Kato, Yuji Joyo, Naoe Tatematsu, Sanshiro Yasuma, Yuko Waguri-Nagaya

**Affiliations:** 1 Orthopedic Surgery, Nagoya City University East Medical Center, Nagoya, JPN; 2 Orthopedic Surgery, Tatematsu Orthopedics and Internal Medicine Clinic, Nisshin, JPN

**Keywords:** acupuncture, extensor digitorum tendon, foot, press-tack needle, tendon rupture

## Abstract

A 30-year-old female patient applied press-tack needles near the left fifth metatarsophalangeal joint to alleviate pain during table tennis. One month later, she presented with impaired dorsiflexion of the left fifth toe. Examination revealed a rupture of the left extensor digitorum longus tendon. Computed tomography was employed to confirm the rupture and associated scar tissue near the fifth metatarsal. Surgical repair revealed synovial hyperplasia, adhesions, and partial tendon degeneration, allowing successful end-to-end repair. Postoperatively, cast immobilization was implemented for four weeks, with a return to table tennis activities one year later. At one year, the patient achieved normal extensor muscle strength and satisfactory toe dorsiflexion and plantarflexion.

To our knowledge, this represents the first reported case of extensor tendon rupture associated with press-tack needle use. Press-tack needles are widely available and often used casually without adequate medical expertise. The needle's placement over the extensor digitorum longus tendon likely led to synovitis, adhesions, and eventual tendon rupture. While press-tack needles are generally associated with minor complications, this case demonstrates that serious adverse events can occur when they are used improperly. This case emphasizes the importance of appropriate acupuncture training and cautious application, particularly near sensitive anatomical structures such as tendons, to prevent such complications.

## Introduction

In sports, acupuncture therapy is frequently employed for purposes such as pain relief, relaxation of muscle tension, improvement of blood flow, and promotion of tissue repair [1]. Intradermal needling using short needles with lengths ranging from 0.6 to 1.2 mm is expected to have sustained effects and effectiveness during play [2,3]. Press-tack needles are widely marketed as simple, minimally invasive devices that can be purchased online and applied without specialized medical training, in contrast to the long needles used in conventional acupuncture, which typically require administration by a trained practitioner.

The dorsum of the foot is anatomically characterized by a thin layer of subcutaneous fat and muscle, beneath which vessels, nerves, and tendons, including the extensor digitorum longus tendon, run in close proximity to the skin surface. This superficial anatomical arrangement renders the region particularly vulnerable to external mechanical stimuli such as needle insertion, pressure, or repetitive microtrauma, and even a device intended for low-intensity, minimally invasive stimulation may pose a mechanical risk to these structures if placed inaccurately. Here, we report a case in which the use of press-tack needles during a sporting activity resulted in rupture of the extensor digitorum longus tendon in the left foot.

## Case presentation

**Figure 1:**
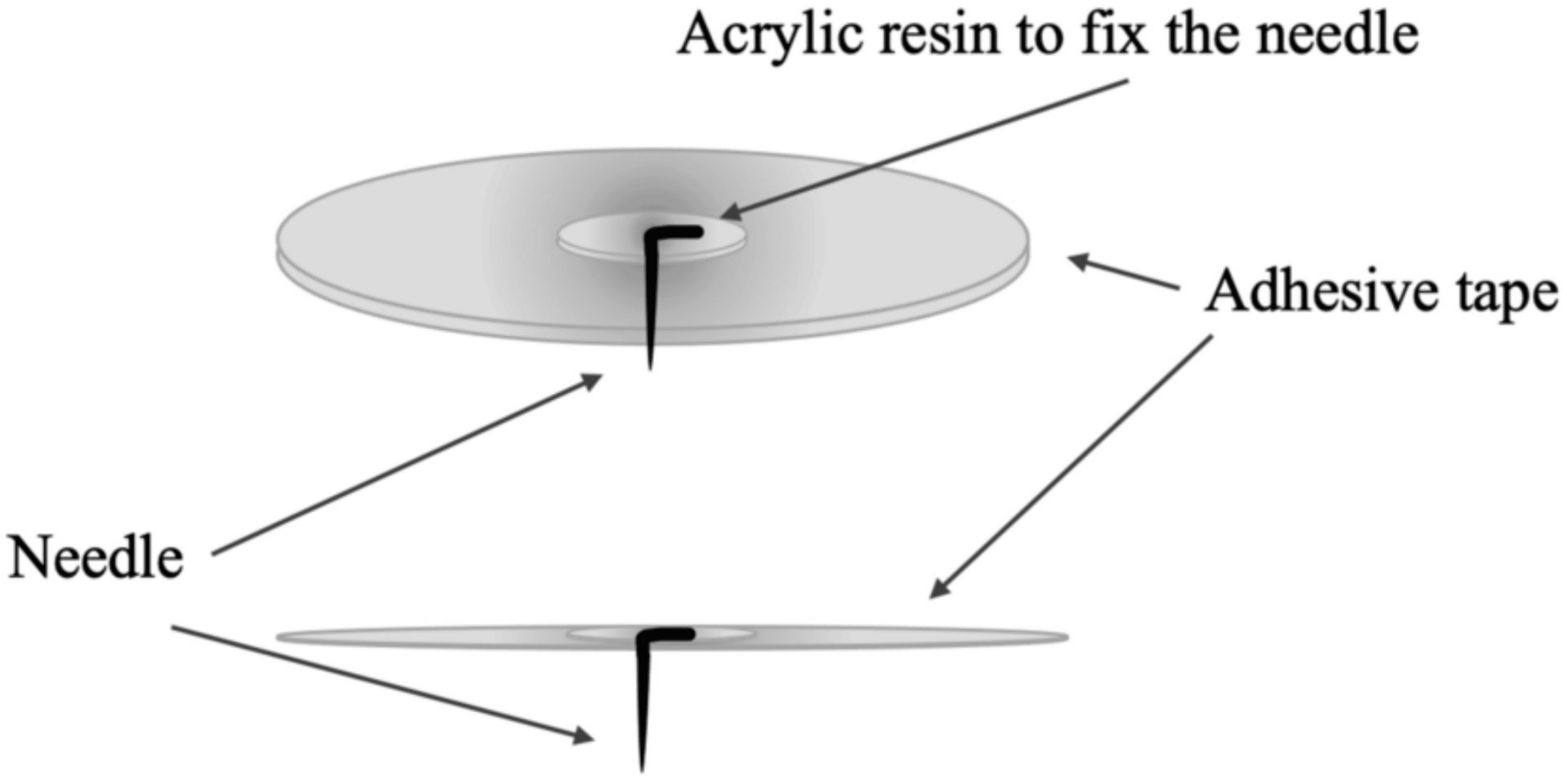
Press tack needle The needle has a diameter of 0.2 mm and a length of 0.6 mm Image credit: This is an original image created by the author Sakurako Kato

**Figure 2:**
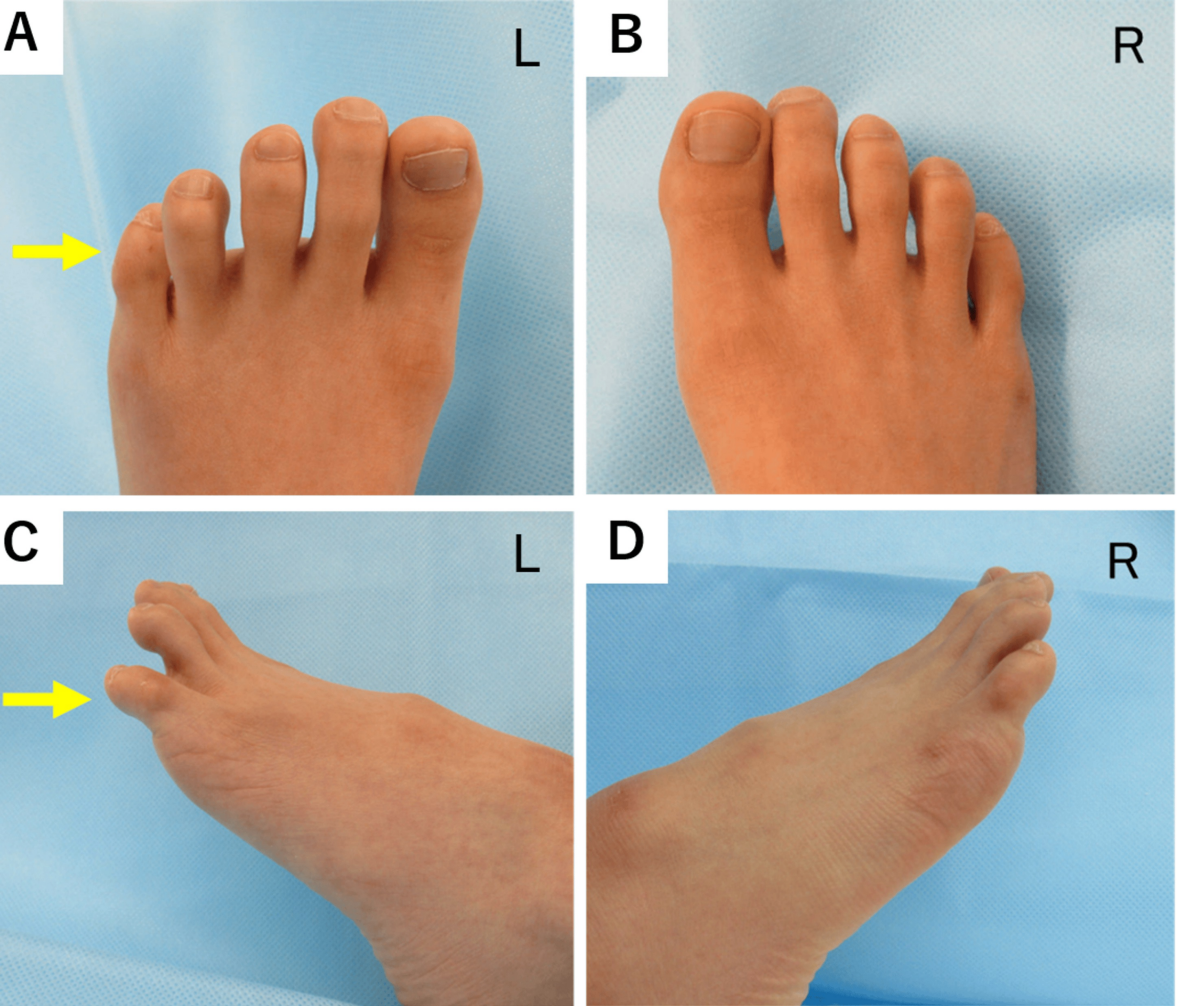
Macrophotographs of the forefoots at the initial visit (A) Dorsal view of the left foot. (B) Dorsal view of the right foot. (C) Lateral view of the left foot. (D) Lateral view of the right foot. Yellow arrows indicate the fifth toe held in a flexed position. Compared with the contralateral side, the left fifth metatarsophalangeal joint was held in a flexed position, and active extension was not possible. The active range of motion was 0° for extension and 65° for flexion. This loss of active extension, in the absence of a fixed contracture or bony deformity on examination, was consistent with extensor tendon rupture

**Figure 3:**
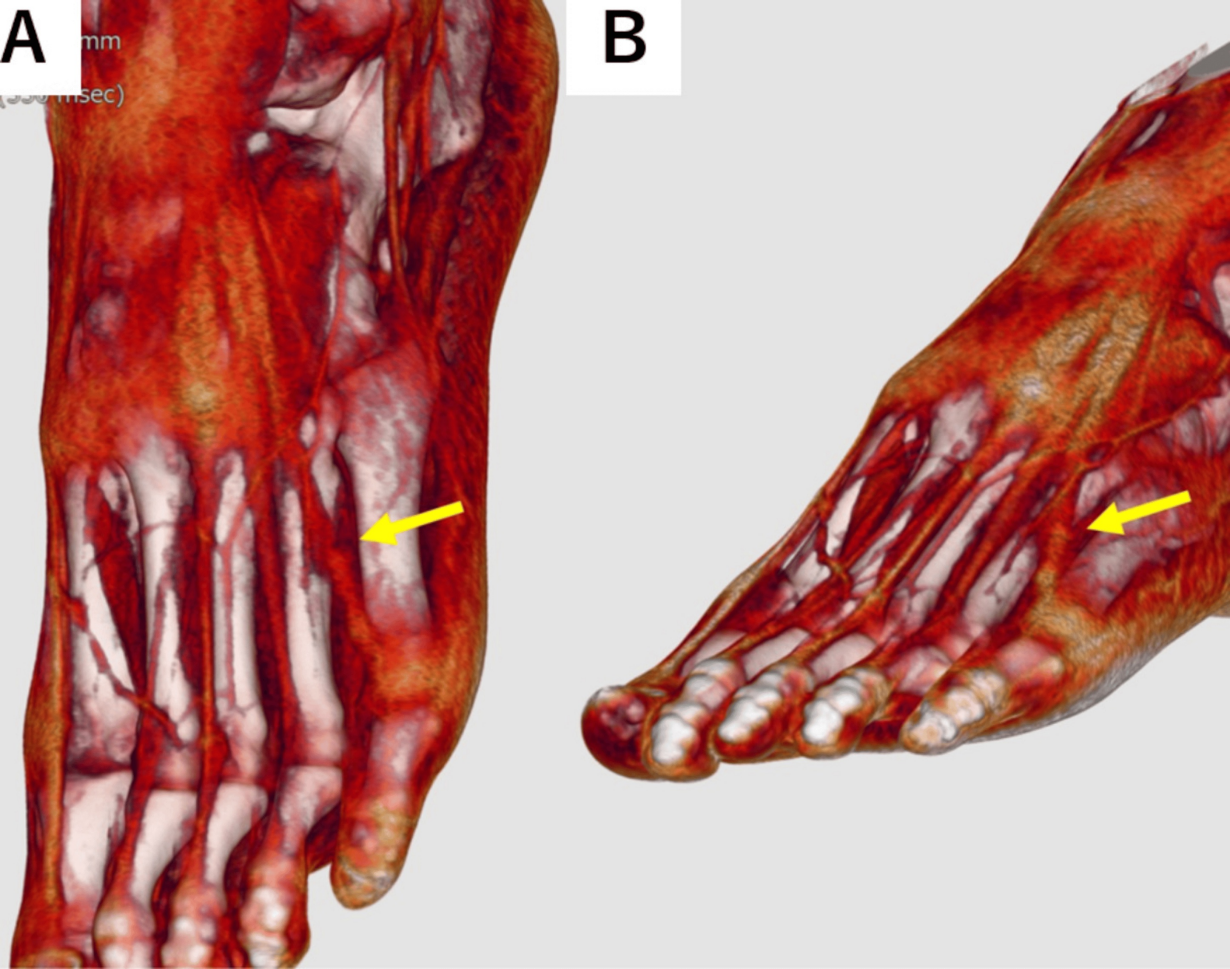
Reconstructed CT images of the left foot highlighting the tendon structures (A) Dorsal view. (B) Oblique view. Yellow arrows indicate the site of rupture and scar tissue in the extensor tendon of the fifth toe. The images suggest a rupture and scar tissue in the left fifth extensor tendon at the level of the fifth metatarsal, indicating a potential tendon rupture in this region CT: computed tomography

A 30-year-old female patient had used press-tack needles (Figure 1) near the left fifth metatarsophalangeal (MTP) joint to alleviate pain experienced during a table tennis match. One month later, she presented with difficulty dorsiflexing the left fifth toe and sought consultation at our department. Examination revealed a rupture of the left extensor digitorum longus tendon (Figure 2). Ultrasound examination failed to clearly visualize the rupture site. MRI was considered an alternative imaging modality; however, it was not pursued due to a several-week scheduling delay at our institution, which was not feasible given the need for timely clinical decision-making. Instead, reconstructed images of the left foot using computed tomography (CT) were obtained. CT was considered particularly useful in this case, as it allows superior assessment of bony structures, including osteophyte formation; osteophytes adjacent to the tendon can act as a mechanical source of chronic friction and are a recognized contributing factor in extensor tendon rupture, making their evaluation clinically relevant in this setting. In addition, CT provided clear evidence of the tendon rupture itself, rendering further imaging with MRI unnecessary for diagnosis and management planning. The CT images suggested a rupture and scar tissue in the left fifth extensor tendon near the level of the fifth metatarsal (Figure 3).

**Figure 4:**
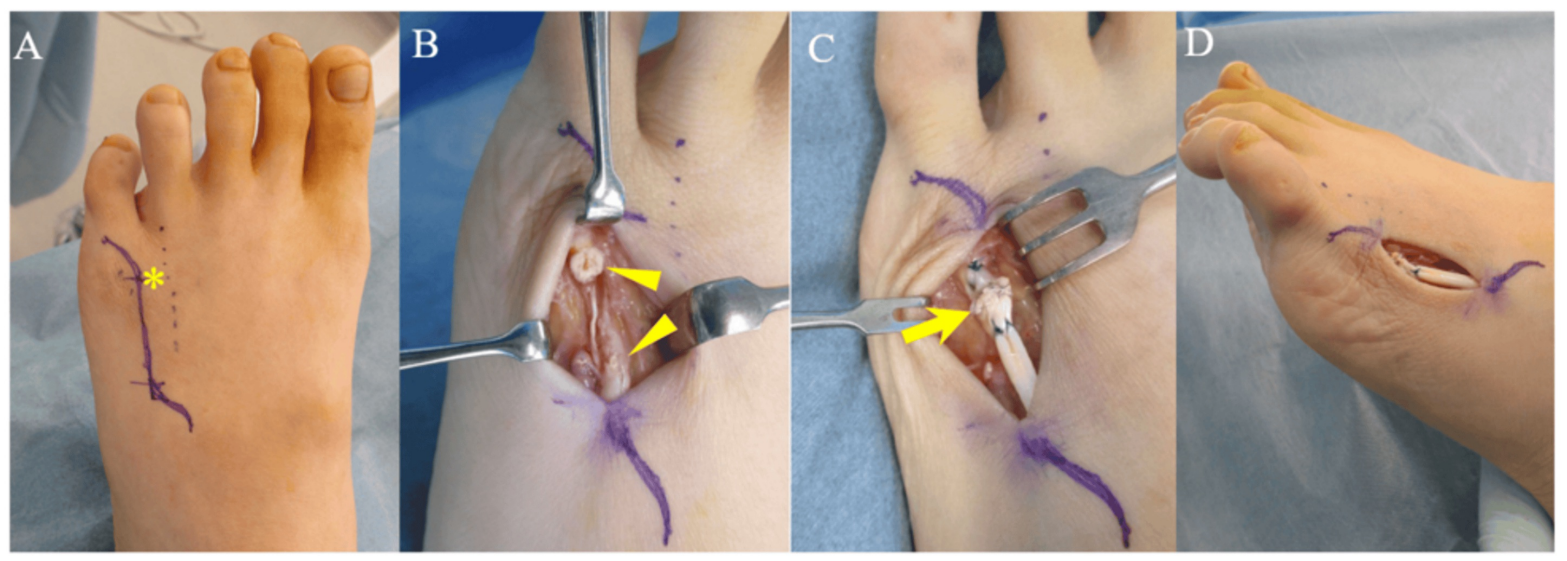
Surgical findings (A) The press tack needling point is marked with an asterisk. (B) Synovial proliferation and adhesions were observed around the ruptured tendon ends (arrow head). (C) Adhesions were dissected, and the degenerated portions of the tendon ends were excised. Upon dorsiflexion of the ankle joint, the proximal tendon stump was mobilized, enabling end-to-end repair (arrow). (D) Tension was evaluated and deemed appropriate, with no signs of rerupture observed when the ankle was positioned in 40° plantar flexion and the fifth MTP joint in 50° flexion MTP: metatarsophalangeal

**Figure 5:**
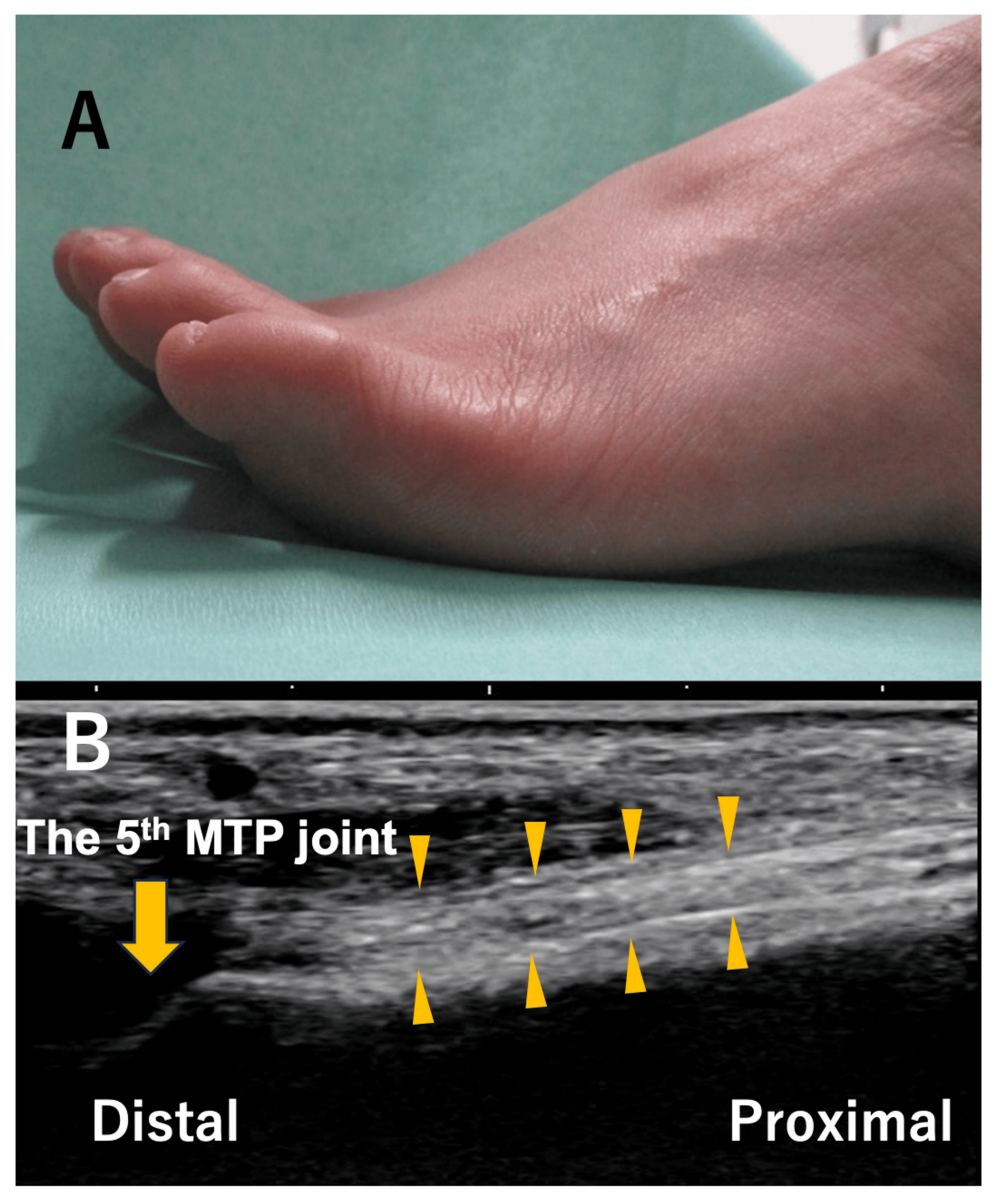
One year postoperatively, (A) extension of the left fifth toe MTP joint was restored, and (B) ultrasonographic evaluation revealed no abnormalities in the extensor tendon contour. The arrowheads indicate the extensor digitorum longus tendon MTP: metatarsophalangeal

Intraoperatively, synovial hyperplasia and adhesions were observed around the rupture site, along with partial tendon degeneration; however, end-to-end repair was feasible (Figure 4). Postoperatively, the patient underwent cast immobilization for four weeks before returning to table tennis activities. One year postoperatively, the active range of motion of the fifth MTP joint was dorsiflexion to 30° and plantarflexion to 65°, and extensor muscle strength was normal (manual muscle testing). Ultrasound examination showed no evidence of rerupture (Figure 5).

## Discussion

In this case, one month had elapsed between the insertion of the press-tack needle acupuncture and the tendon rupture, during which synovitis and adhesions with surrounding tissues developed, complicating ultrasound imaging. Reconstructing the tendon structures using CT confirmed the rupture, underscoring the utility of CT in diagnosing chronic extensor tendon ruptures.

The press-tack needle used in this case consisted of a small, fine needle embedded in a circular adhesive tape, as shown in Figure 1. This device is designed to be applied directly to the skin, where it remains in place, providing continuous, low-intensity stimulation to specific acupuncture points. It is favored for its ease of use, minimal invasiveness, and sustained therapeutic effects in managing pain, inflammation, and other conditions. However, press-tack needles are readily available online and are sometimes used casually without proper medical knowledge.

"Ashirinkyū" or "Zúlínqì" is an acupuncture point in traditional Chinese and Japanese medicine, corresponding to the Gallbladder meridian (Gallbladder 41) in the acupuncture meridian system [4]. It is located on the dorsum of the foot, in the depression distal to the junction of the fourth and fifth metatarsal bones, near the lateral side of the extensor digitorum longus tendon. This point is commonly employed for therapeutic effects on disorders related to the head, eyes, and chest, as well as for treating pain and stiffness in the foot. However, accurate positioning can be challenging for nonexperts. In this case, the press-tack needle had been placed directly over the extensor digitorum longus tendon, and this anatomical misplacement is considered a plausible contributing factor to the subsequent tendon rupture, although a definitive causal relationship cannot be established from a single case.

Other etiologies for extensor tendon rupture, such as repetitive mechanical microtrauma from sports activity or degenerative tendinopathy, should also be considered. The patient did engage in sports activity; however, this was limited to a recreational level with low frequency and was therefore considered insufficient to generate the cumulative repetitive microtrauma typically implicated in overuse-related tendon rupture. Furthermore, the rupture developed at the precise anatomical site of needle insertion within a temporally consistent interval. This close spatial and temporal correlation makes needle-related mechanical injury a more plausible explanation than an unrelated degenerative or overuse etiology in this patient. Nonetheless, we acknowledge that, in the absence of histopathological confirmation, the possibility of a coincidental or multifactorial process cannot be entirely excluded.

Systematic reviews have reported common adverse events associated with acupuncture, including pneumothorax, syncope, subarachnoid hemorrhage, and infections [5-7]. These complications are typically associated with the use of long needles used in conventional acupuncture. In contrast, complications related to press-tack needle acupuncture have generally been limited to minor adverse events such as bleeding, hematoma, and pain in several randomized studies [3,8]. To our knowledge, tendon rupture has not previously been reported as a complication of press-tack needle acupuncture, and this case therefore represents a novel report highlighting a previously unrecognized risk associated with this device.

Liew and Teh reported a flexor pollicis longus tendon rupture following traditional-needling acupuncture for trigger thumb, performed by an acupuncturist [9]. This prior report, however, involved a long needle inserted by a trained acupuncturist, which differs mechanically and in usage context from the fine, superficial press-tack needle used in the present case. We therefore do not draw a direct causal comparison between the two reports; rather, we cite this report to illustrate that tendon injury, although rare, has been documented in association with acupuncture needling more broadly. Liew and Teh emphasized that acupuncture training should be closely monitored by health authorities and that meticulous care is essential when needling at high-risk traditional acupuncture points, particularly in areas with delicate neurovascular and tendon structures [9].

Press-tack needles are widely marketed as simple, minimally invasive devices that can be purchased online and applied without specialized medical training, and they are generally regarded as carrying a low risk of serious adverse events [3,8]. However, the present case suggests that even this seemingly low-risk device can, when misapplied over anatomically vulnerable structures such as a tendon, result in serious complications, including tendon rupture. This finding indicates that the ease of use and accessibility of press-tack needles should not be equated with an absence of risk and highlights the importance of anatomical knowledge and caution, even among lay users, when applying these devices at acupuncture points located near tendons or other delicate structures.

## Conclusions

We report a rare case suggesting a possible association between press-tack needle acupuncture and extensor tendon rupture in the toe during sports activity. Given the single-case nature of this report and the absence of histopathological confirmation, this should be regarded as a possible, rather than an established complication, and further accumulation of cases is needed to clarify causality. Nonetheless, sports medicine practitioners should be aware of this potential complication when advising patients on the use of press tack needles near tendinous structures, and patients should be counseled on proper needle placement and the importance of seeking specialized guidance before self-applying these devices during athletic activity.

## References

[1] Pujalte GG, Malone M, Mandavalli A, Phrathep DD, Shah NP, Perlman AI (2023). Acupuncture in sports medicine. J Acupunct Meridian Stud.

[2] Okuma Y, Yoshida N, Miyazaki S, Hisajima T, Takahashi H, Miyakawa S (2016). Effects of stimulation with press tack needle acupuncture on muscle fatigue. Jap Acup Mox.

[3] Schröder S, Meyer-Hamme G, Friedemann T (2017). Immediate pain relief in adhesive capsulitis by acupuncture-a randomized controlled double-blinded study. Pain Med.

[4] Yano T, Oyama Y, Yamada N, Mori K, Yukimachi T (1990). Specificity of meridian and acupuncture-points: relationship between acupuncture-points of the gallbladder meridian on the lower extremity and gallbladder's form. J Jap Soc Bal Clim Phys Med.

[5] Chan MW, Wu XY, Wu JC, Wong SY, Chung VC (2017). Safety of acupuncture: overview of systematic reviews. Sci Rep.

[6] Zhang J, Shang H, Gao X, Ernst E (2010). Acupuncture-related adverse events: a systematic review of the Chinese literature. Bull World Health Organ.

[7] Kim TH, Lee MS, Birch S, Alræk T, Norheim AJ, Kang JW (2023). Publication status and reporting quality of case reports on acupuncture-related adverse events: a systematic reviews of case studies. Heliyon.

[8] Maurer J, Friedemann T, Chen Y (2024). A randomized controlled study on acupuncture for peri-operative pain after open radical prostatectomy. BJU Int.

[9] Liew SK, Teh KK (2021). Flexor pollicis longus rupture following acupuncture for trigger thumb: a case report. Acupunct Med.

